# Nrf3 alleviates oxidative stress and promotes the survival of colon cancer cells by activating AKT/BCL-2 signal pathway

**DOI:** 10.1515/biol-2022-0790

**Published:** 2023-11-23

**Authors:** Bi-Qing Cai, Wan-Meng Chen, Meng-Wei Chen, Ya-Hui Chen, Jian-Cai Tang

**Affiliations:** Institute of Basic Medicine and Forensics Medicine, North Sichuan Medical College, Fu Jiang Road 234, Shunqing District, Nanchong, Sichuan, 637000, China; Key Laboratory of Metabolic Drugs and Biological Products, Nanchong, China

**Keywords:** Nrf3, oxidative stress, colon cancer, Akt/Bcl-2, cell proliferation, apoptosis

## Abstract

Oxidative stress is closely linked to tumor initiation and development, conferring a survival advantage to cancer cells. Therefore, understanding cancer cells’ antioxidant molecular mechanisms is crucial to cancer therapy. In this study, we discovered that H_2_O_2_-induced oxidative stress increased Nrf3 expression in colon cancer cells. Overexpression of Nrf3 decreased H_2_O_2_-mediated cytotoxicity and apoptosis. Furthermore, Nrf3 reduced reactive oxygen species levels and malondialdehyde concentrations after H_2_O_2_ treatment. Mechanistically, H_2_O_2_-mediated cell apoptosis involves multiple signaling proteins, including Akt, bcl-2, JNK, and p38. An increase in Nrf3 expression in colon cancer cells treated with H_2_O_2_ partly reversed Akt/Bcl-2 inhibition, whereas it decreased activation of p38 and JNK. In addition, we found that increasing Nrf3 decreased stress-associated chemical-induced cell death, resulting in drug resistance. According to these results, Nrf3 is critical for drug resistance and oxidant adaptation.

## 1 Introduction

A colorectal cancer diagnosis is the third most common cancer in the world and the fourth leading cause of cancer-related death, responsible for 9.2% of all deaths [1]. Over the last three decades, there has been a consistent rise [2] in the prevalence of early-onset CRC among individuals under the age of 50. Colorectal cancer [3] is currently treated primarily with surgery, radiotherapy, and chemotherapy. Despite significant progress in treatment modalities and management strategies, survival rates of patients have not improved significantly. Consequently, further investigation of colorectal cancer molecular mechanisms and biological behaviors is essential to develop new therapeutic strategies.

Several factors [4] are responsible for the development of colorectal cancer, such as genetic factors, gene mutations, and abnormal signal pathway activation, Oxidative stress [5] induced DNA damage and protein conformations such as DNA single-strand or double-strand breaks, and protein misfolding, which is regarded as a key factor for the initiation and progression of colorectal cancer. Oxidative stress [6] refers to increased intracellular reactive oxygen species (ROS) levels due to an imbalance between oxidants and antioxidants. The elevated levels of ROS observed in cancer cells compared to normal cells can be attributed, in part, to augmented metabolic activity and impaired mitochondrial function [7]. The role of ROS [8,9] in tumor initiation and development is two sided. Low and intermediate ROS levels promote cancer cell proliferation and survival, whereas excessive ROS induces cancer cell apoptosis. The varying levels of ROS play a crucial role in the formulation of effective anticancer approaches aimed at modulating ROS levels. In response to oxidative stress, cancer cells enhance their antioxidant capacity as a means of adapting, consequently leading to the development of drug resistance [10]. However, several drugs [11,12,13], such as cisplatin, 5-Fu, bleomycin, induce cancer cell death by increasing ROS levels. Thus, understanding the mechanisms underlying oxidative adaptation may be critical for cancer therapy.

Nuclear factor erythroid 2-like-3 (Nrf3) [14] belongs to the cap ‘n’ collar family, together with Nrf1, Nrf2, Bach1, and Bach2, all of which have a similar domain. Several reports [15,16,17] have shown that Nrf2 plays a critical role in tumor initiation and development. Both Nrf3 and Nrf2 [18] exhibit identical characteristics to the Neh1L, Neh3L, Neh5L, and Neh6L sequences found in the source’s structural sequence. Additionally, Nrf2 possesses homologous regions known as Neh2 and Neh4. The N-terminal region of Nrf3 is 150 amino acids longer than that of Nrf2, and this sequence includes an endoplasmic reticulum anchoring signal, resulting in distinct subcellular localization compared to Nrf2. However, Nrf3’s role in cancer remains elusive, partly because Nrf3-deficient mice exhibit no evident abnormalities [14]. Recently, reports [19,20,21] have displayed that Nrf3 may also be involved in tumor initiation, progression, and oxidative stress. Multiple types of cancer cells exhibit Nrf3 influence proliferation, survival, migration, and chemoresistance of cancers such as breast cancer [22], colon cancer [20,23], and hepatocellular carcinoma [24]. Nrf3 may also combine with the antioxidant response element (ARE) to promote antioxidant gene expression. Therefore, further exploration of the function of Nrf3 in oxidative stress may be significant for cancer therapy.

In the study, the role of Nrf3 in H_2_O_2_-induced oxidative stress colon cancer cells was investigated. As a result of H_2_O_2_, Nrf3 expression increased in a dose-dependent manner. Over-expression of Nrf3 upregulated colon cancer cell survival and decreased apoptosis under H_2_O_2_-induced oxidative stress. Furthermore, Nrf3 promoted the activation of Akt and Bcl-2, attenuating the activity of p38 and JNK. Interestingly, Nrf3 decreased 5-Fu inducing cell death. Based on these results, Nrf3 may play a crucial role in oxidant adaptation and provide a novel target for treating colon cancer.

## 2 Materials and methods

### 2.1 Cell culture and reagents

HCT116, HT29, and SW620 cells were purchased from CellBio Company (Shanghai, China) and cultured with RPMI‑1640 medium (Gibco; Thermo Fisher Scientific, Inc.), supplemented with 10% fetal bovine serum (FBS; cat. no. C04001-500; viva cell; Germany), in 5% CO_2_ at 37°C. Experiments employ cells that have undergone a maximum of five passages. The Cell Counting Kit-8 (CCK-8) was purchased from Beyotime (catalog number: C0038, Shanghai, China). The Cell Apoptosis Kit (catalog number: AD10-10, Kyushu, Japan) was purchased from Dojindo.

### 2.2 Chemicals and treatment

H_2_O_2_ (30%) was purchased from Sigma (catalog number: 7722-84-1), and 5-Fu was provided by the Selleck Company (catalog number: S1209). H_2_O_2_ (30%) was diluted with DMEM to final concentrations (200, 400, 600 μM), and 5-Fu was dissolved in DMSO to 100 mM stock solution. The compound 5-FU was subjected to dilution at various concentrations (1.5, 3, 6, 12, 24 μM) to administer treatment to cells over a duration of 12 h.

### 2.3 Construction of colon cancer cell line for stable Nrf3 overexpression and knock-down

The recombinant lentivirus for the overexpression of Nrf3 was purchased from Santa Cruz Biotechnology (catalog number: sc-404543-LAC). Two shRNA target sequences for Nrf3 were designed and inserted into the pLKO.1 vector. Recombinant lentiviruses were generated through co-transfecting shRNA plasmids. The Nrf3 shRNA interference target sequences were in accordance with our previous study [25].

A quantity of 2 × 10^5^ HCT116 cells were initially placed in each well and allowed to grow until reaching 40% confluency. Alongside the complete medium, lentiviral particles were introduced at a final concentration of polybrene (5 µM). Following thorough mixing, the original medium was substituted, and infection was permitted for a duration of 24 h. Subsequently, the medium was replaced once more, and puromycin hydrochloride was introduced to facilitate screening for a period of 48 h. The impact of Nrf3 overexpression and knockdown was assessed through the utilization of western blotting.

### 2.4 CCK-8 assay

HCT116 and HCT116/Nrf3 cells were seeded into 96-well plates and cultured overnight. H_2_O_2_ (0, 200, 400, and 600 μM) and 5-FU (0, 15, 3, 6, 12, and 24 μM) were, respectively, applied to HCT116 and HCT116/Nrf3 cells for 12 h. A CCK-8 test was used to determine the viability of cells according to the manufacturer’s instructions.

### 2.5 Flow cytometry

HCT116 and HCT116/Nrf3 cells were seeded in 6-well plates at 4 × 10^5^ cells/well. The cells were treated with different concentrations of H_2_O_2_ (0, 200, 400, and 600 μM) for 12 h. Cell apoptosis was determined using flow cytometry, according to the manufacturer’s instructions. Briefly, HCT116 or HCT116/Nrf3 cells were harvested by trypsin without EDTA. The cells were then resuspended in a binding buffer, which consisted of 2.5 µL AnnexinV FITC and 2.5 µL PI, and incubated for 30 min in the absence of light. The percentage of apoptotic cells was determined using a Beckman Flow Cytometer (Beckman Coulter, Inc.) employing a count model, and the data were analyzed using FlowJo software (Version 10.0; FlowJo LLC).

### 2.6 The determination of ROS levels and malondialdehyde (MDA) concentrations

Cells were seeded into six-well plates at a density of 5 × 10^5^ cells/well. Subsequently, the cells were exposed to varying concentrations of H_2_O_2_ (200, 400, and 600 μM) for a duration of 12 h. The production of ROS in colon cancer cells was evaluated using 2′,7′-dichlorofluorescein diacetate (DCFH-DA; Beyotime, Shanghai, China, catalog number: S0033M). DCFH-DA was diluted with a serum-free medium at a ratio of 1:1,000, resulting in a final concentration of 10 μmol/L. The cell culture medium was then replaced with DCFH-DA solution, and the cells were incubated at 37°C for 20 min. Following incubation, the cells were washed three times with a serum-free medium. Flow cytometry (BD LS) was employed to analyze the levels of ROS.

Intracellular lipid peroxidation was assessed through the quantification of MDA concentration using a Lipid Peroxidation MDA Assay Kit (Jiancheng, Nanjing, China, catalog number: A003-1-2). Cells were subjected to various concentrations of H_2_O_2_ (200, 400, and 600 μM) for a duration of 12 h. Subsequently, a specific quantity of cells was collected and the lysate was incubated at 4°C for 2 h, followed by centrifugation at 12,000 rpm for 10 min to obtain the supernatant for subsequent analysis. Next, 0.2 mL of MDA detection working solution was added, and the mixture was thoroughly mixed before being heated to 100°C or boiled in a water bath for 15 min. The absorbance was then measured at 532 nm with a microplate reader. After the MDA content in the sample solution was calculated, it was normalized to the MDA content in the parental cell sample as the protein content per unit weight.

### 2.7 Western blotting

A six-well plate was plated with colon cancer cells and treated with H_2_O_2_. Then, the cells were collected, and total proteins were extracted by RIPA lysis buffer (Beyotime Institute of Biotechnology) and concentration was measured by BCA kit (Beyotime Institute of Biotechnology). For electrophoresis, 20 µg of protein samples were loaded in each lane and were separated using 10% SDS-PAGE gels. Then, the proteins in the gels were transferred onto PVDF membranes, which were then incubated in a blocking buffer (3% bovine serum albumin; Beijing Solarbio Science & Technology Co., Ltd.) for 1 h at room temperature. Next, membranes were blocked with primary antibodies at 4°C overnight, including anti-Nrf3 (cat. no. PA5-102015; 1:1,000; Thermo Fisher Scientific, Inc.), anti-phospho-Akt (S473) (cat. no. 4060; 1:1,000; Cell Signaling Technology, Inc.), anti-Akt (cat. no. 9272; 1:1,000; Cell Signaling Technology, Inc.), anti-Bcl-2 (cat. no. 4223S; 1:1,000; Cell Signaling Technology, Inc.), anti-P38 (cat. no. 14451; 1:1,000; Cell Signaling Technology, Inc.), anti-phospho-P38 (cat. no. 4511; 1:1,000; Cell Signaling Technology, Inc.), anti-JNK (cat. no. 9252; 1:1,000; Cell Signaling Technology, Inc.), anti-phospho-JNK (cat. no. 4668; 1:1,000; Cell Signaling Technology, Inc.), and anti-GAPDH (cat. no. AB0036; 1:5,000; Abways Technology) antibodies. The following day, the membranes were incubated with the relevant secondary antibody. Between and after incubation with the antibodies, membranes were washed with TBST. Signals were visualized using Immobilon ECL Ultra Western HRP (cat. no. WBULS0100, MilliporeSigma). The results were analyzed using a ChemiDoc XRS + Gel Imaging System (Bio-Rad Laboratories, Inc)

### 2.8 Statistical analysis

All data are presented as mean ± standard deviation. Comparisons between the two groups were made using Student’s *t*-tests. Three or more groups were compared using a one-way analysis of variance with Tukey’s post hoc test. Differences were considered statistically significant at *P* < 0.05. All experiments were repeated three times.

## 3 Results

### 3.1 H_2_O_2_-mediated oxidative stress increased the expression of Nrf3 in colon cancer cells

**Figure 1:**
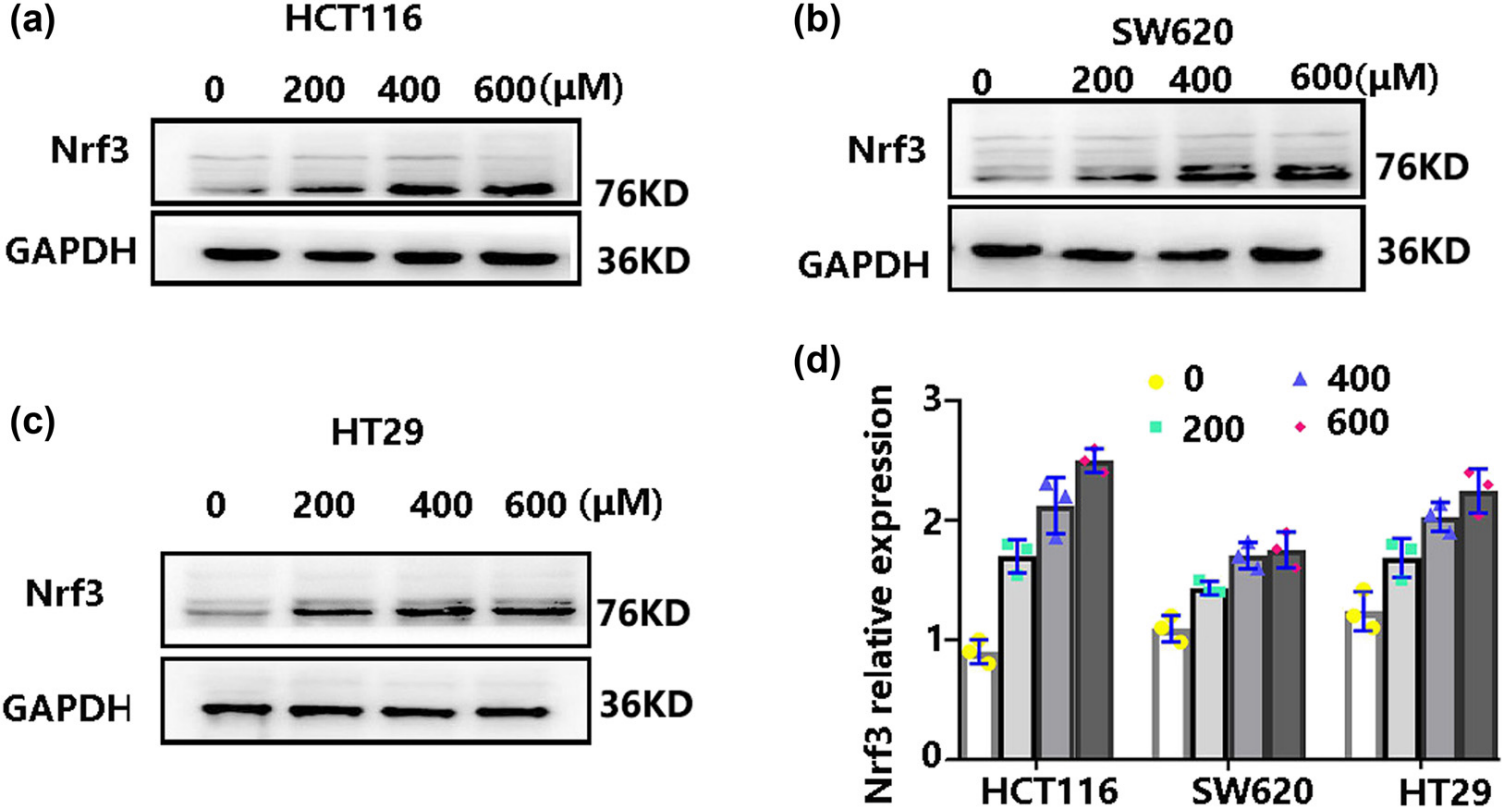
H_2_O_2_ upregulates Nrf3 expression. Colon cancer cells were subjected to various concentrations of H_2_O_2_ for a duration of 12 h. Following this, protein extraction was performed and Nrf3 expression was detected using Western blot analysis. (a) The expression of Nrf3 in HCT116 cells after H_2_O_2_ treatment. (b) The expression of Nrf3 in SW620 cells after H_2_O_2_ treatment. (c) The expression of Nrf3 in HT29 cells following H_2_O_2_ treatment. (d) The relative quantification of Nrf3 expression (**p < 0.01). The results indicate that H_2_O_2_ upregulates Nrf3 expression. All experiments were conducted in triplicate.

In the initial set of experiments, we examined the effect of Nrf3 expression induced by H_2_O_2_ in colon cancer cells. To address this, the cells were cultured in 6-well plates with a density of 5 × 10^5^ cells/well and subsequently exposed to different concentrations of H_2_O_2_: 200, 400, and 600 μM. Following a 12-h incubation period at 37°C, the cells were collected and their total protein content was extracted. The expression of Nrf3 was assessed using Western blot analysis. The findings revealed a dose-dependent elevation in Nrf3 levels in HCT116, HT29, and SW620 cells upon exposure to H_2_O_2_ (Figure 1a–d). Notably, the most substantial increase in Nrf3 expression was observed in HCT116 cells treated with 400 μM H_2_O_2_. All experiments were repeated three times.

### 3.2 Over-expression Nrf3 decreased H_2_O_2_-induced cell death

**Figure 2:**
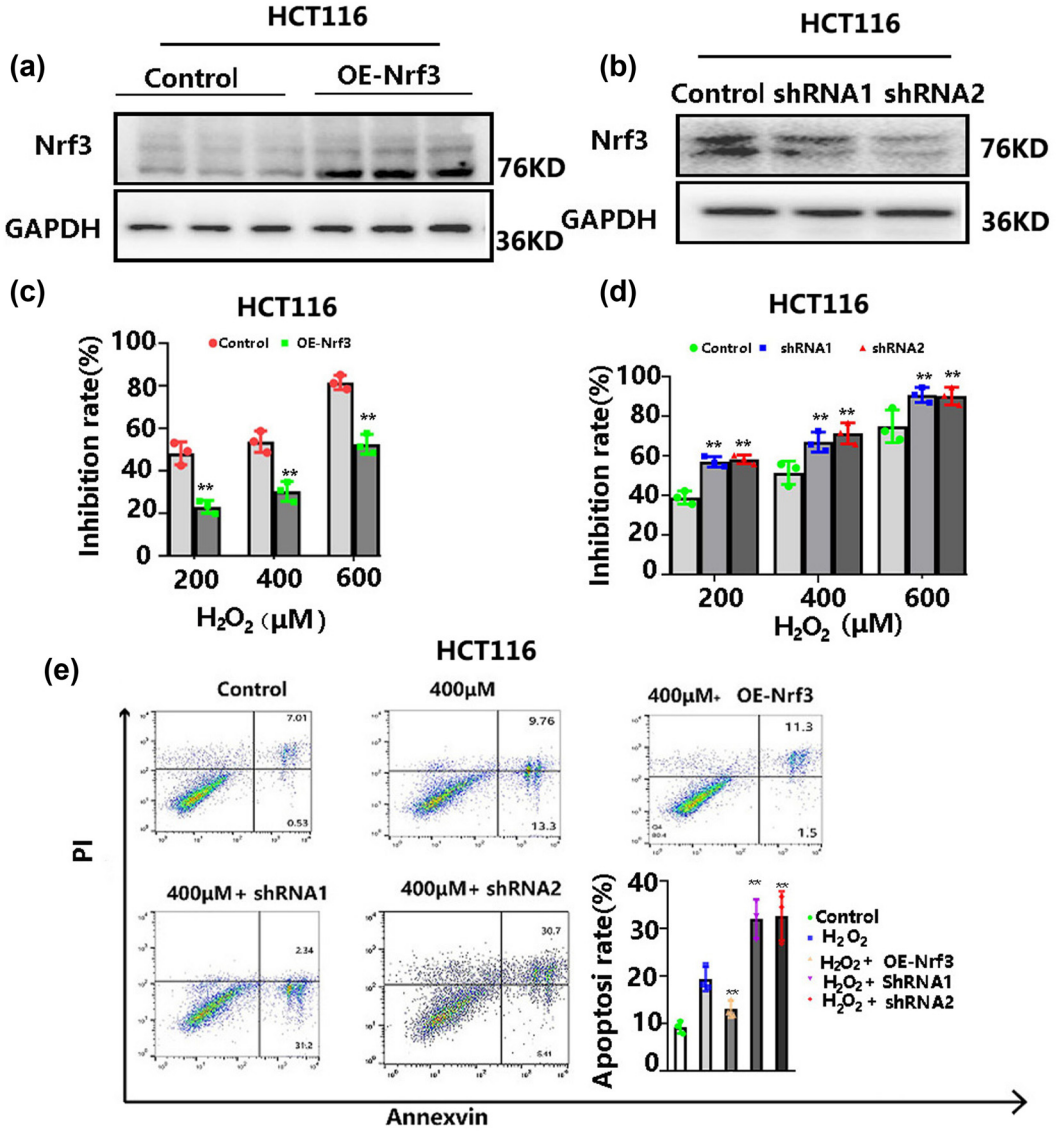
Nrf3 decreases H_2_O_2_-induced cell death. Specifically, the study investigates the effects of overexpressing Nrf3 in HCT116 cells (a) and knocking down Nrf3 in HCT116 cells (b). The results indicate that overexpression of Nrf3 reduces the inhibition rate of colon cancer cells following H_2_O_2_ treatment (c), while knockdown of Nrf3 increases the inhibition rate of colon cancer cells (d). Furthermore, the study examines the influence of Nrf3 on cell apoptosis under varying concentrations of H2O2 (e).

To explore the effect of Nrf3 in H_2_O_2_-induced cell death, we constructed cells over-expressing Nrf3 (HCT116/Nrf3)and cells with knocked down Nrf3 (HCT116/ShNrf3-1 and HCT116/ShNrf3-2). The effect of over-expression and knock-down Nrf3 was determined using Western blotting (Figure 2a and b). Cells were treated with different concentrations of H_2_O_2_ (0, 200, 400, and 600 μM) for 12 h. CCK-8 was used to determine cell viability. Compared with HCT116 cells, the inhibition rate for HCT116/Nrf3 cells was evidently decreased after H_2_O_2_ treatment (Figure 2c). Knock-down of Nrf3 had the opposite effect (Figure 2d). Cell apoptosis was determined using flow cytometry as described in Section 2.5. The results showed that over-expression of Nrf3 reduced cell apoptosis, and Nrf3 knock-down had the opposite results (Figure 2e). These data displayed that Nrf3 over-expression reduces the cytotoxicity of H_2_O_2_-mediated oxidative stress, indicating that Nrf3 promotes cell survival under oxidative stress.

### 3.3 Nrf3 decreased the intracellular ROS and MDA under H_2_O_2_-induced oxidative stress in HCT116

To investigate the possible mechanisms by which Nrf3 reduces H_2_O_2_-induced cytotoxicity, we determined the concentration of ROS and MDA after H_2_O_2_ treatment as described in Section 2.6.

**Figure 3:**
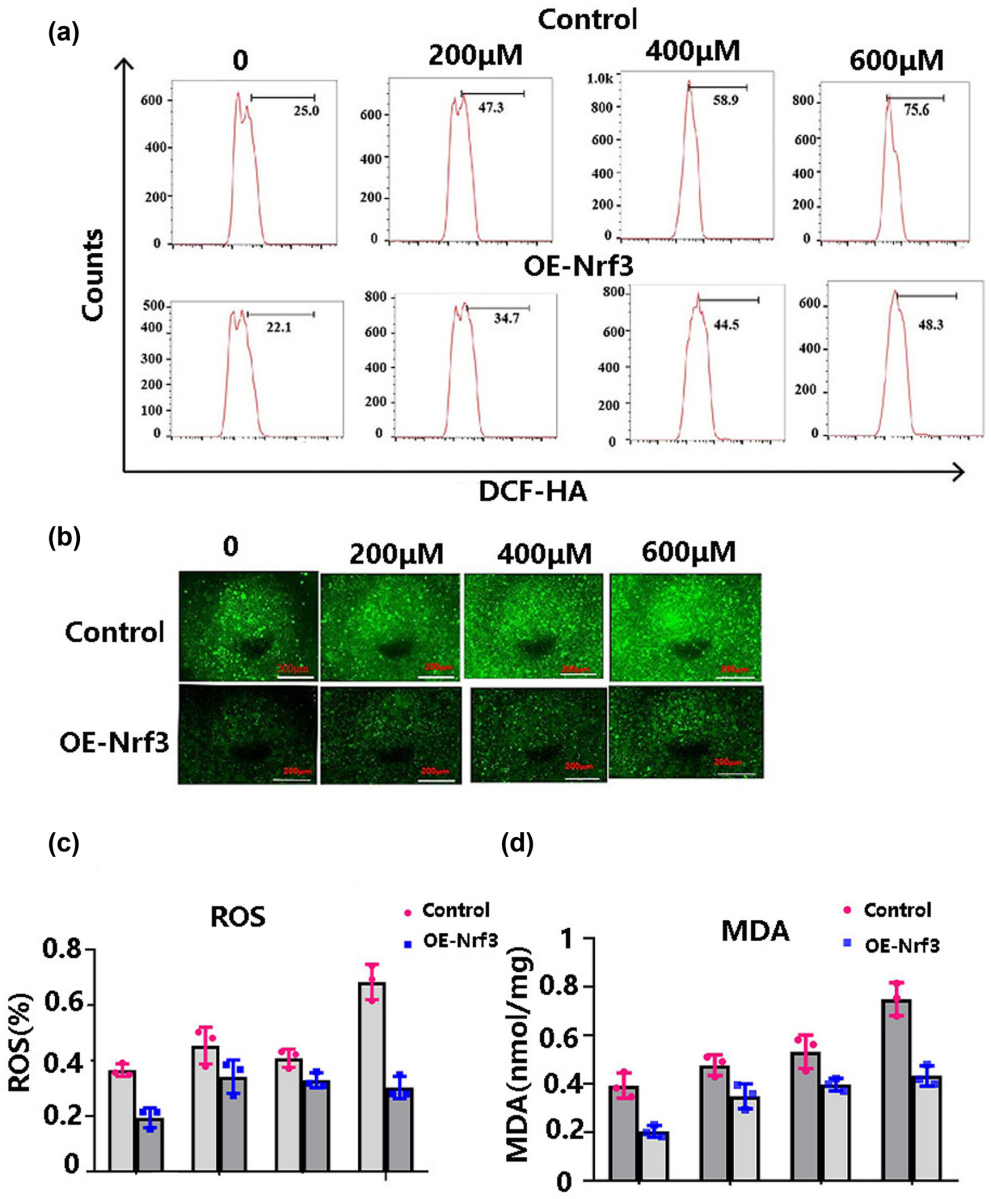
The impact of Nrf3 on the reduction of ROS and MDA levels. (a) The typical images of flow cytometry, (b) the representative fluorescence graphs, (c) the proportion of docosahexaenoic acid following different concentrations of hydrogen peroxide (H_2_O_2_), and (d) the concentration of MDA.

In comparison to HCT116 cells, the concentration of ROS in HCT116/Nrf3 cells exhibited a decrease subsequent to treatment with varying concentrations of H_2_O_2_ (0, 200, 400, and 600 μM) as depicted in Figure 3a–c. Additionally, the levels of MDA were assessed, revealing that Nrf3 diminished the intracellular MDA concentration following H_2_O_2_ treatment, as shown in Figure 3d. These findings suggest that Nrf3 potentially counteracts ROS to reinstate the cellular redox equilibrium and facilitate the survival of cancer cells.

### 3.4 H_2_O_2_-mediated oxidative stress involves multiple signal pathways in colon cancer cells

Oxidative stress-mediated cell apoptosis may involve multiple signaling pathways, including pro-survival or pro-death signals. In our experiments, the known molecules were selected to explore the possible mechanisms of Nrf3-decreased cytotoxicity of H_2_O_2_, which included Akt, Bcl-2, P38, and JNK. These molecules are critical for maintaining cell survival (Akt and Bcl-2) and promoting cell apoptosis (JNK and p38) in the oxidative stress of cancer cells.

**Figure 4:**
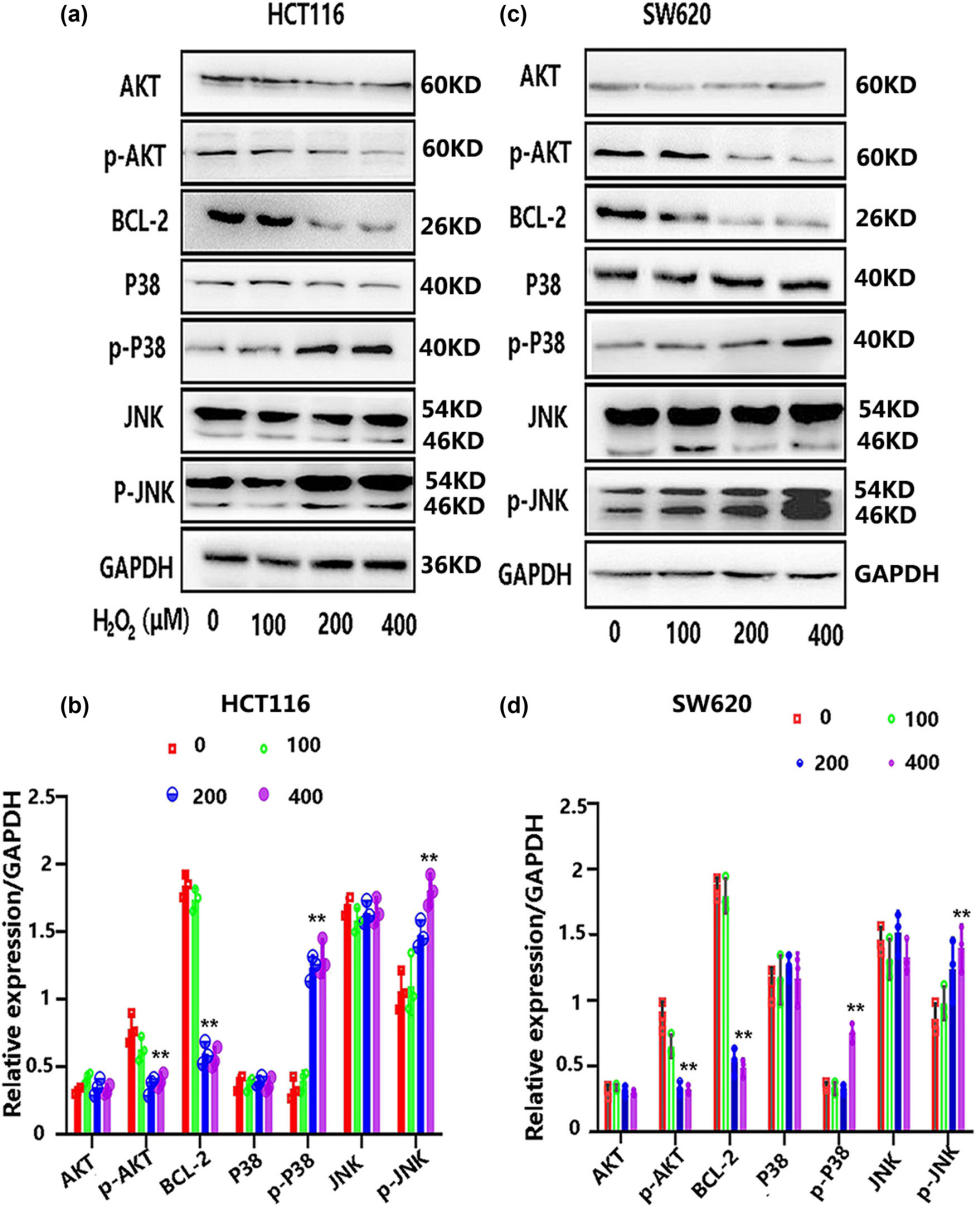
The involvement of multiple cell signals in H_2_O_2_-mediated oxidative stress. HCT116 and SW620 cell lines were subjected to various concentrations of H_2_O_2_ for a duration of 12 h, and subsequent protein extraction was performed to assess alterations in cell signals. Specifically, the expression levels of Akt, p-Akt, Bcl-2, JNK, p-JNK, P38, and p-P38 were evaluated in HCT116 cells (a and b), as well as in SW620 cells (c and d), following exposure to different concentrations of H_2_O_2_.

The cells were placed into six-well dishes with a density of 5 × 10^5^ cells/well. Afterward, the cells were subjected to different levels of H_2_O_2_ (200, 400, and 600 μM) for 12 h. The related molecules were determined by Western blot. The data showed that the expression of p-Akt and Bcl-2 was reduced by treatment with 200 and 400 μM H_2_O_2_ but not by 100 μM H_2_O_2_ in HCT116 and SW620 cells. Phosphorylation levels of JNK and P38 were significantly boosted by H_2_O_2_ treatment at 200 and 400 μM but not 100 μM in both cell lines (Figure 4a–d). These results displayed that high levels of H_2_O_2_ (400 μM) suppressed pro-survival signals (Akt and Bcl-2) while enhancing pro-apoptosis signals (JNK and p38).

### 3.5 Over-expression Nrf3 regulates the oxidative signals under H_2_O_2_-mediated oxidative stress

Given the observed decrease in H_2_O_2_-induced cytotoxicity as a result of Nrf3, our subsequent investigation aimed to ascertain the potential involvement of Nrf3 in the regulation of oxidative signals within colon cancer cells under conditions of H_2_O_2_-mediated oxidative stress. The cells were allocated into six-well plates at a density of 5 × 10^5^ cells/well. Subsequently, the cells were exposed to varying concentrations of H_2_O_2_ (200, 400, and 600 μM) for a duration of 12 h. The proteins were extracted and subsequently analyzed for the presence of related molecules using Western blotting.

**Figure 5:**
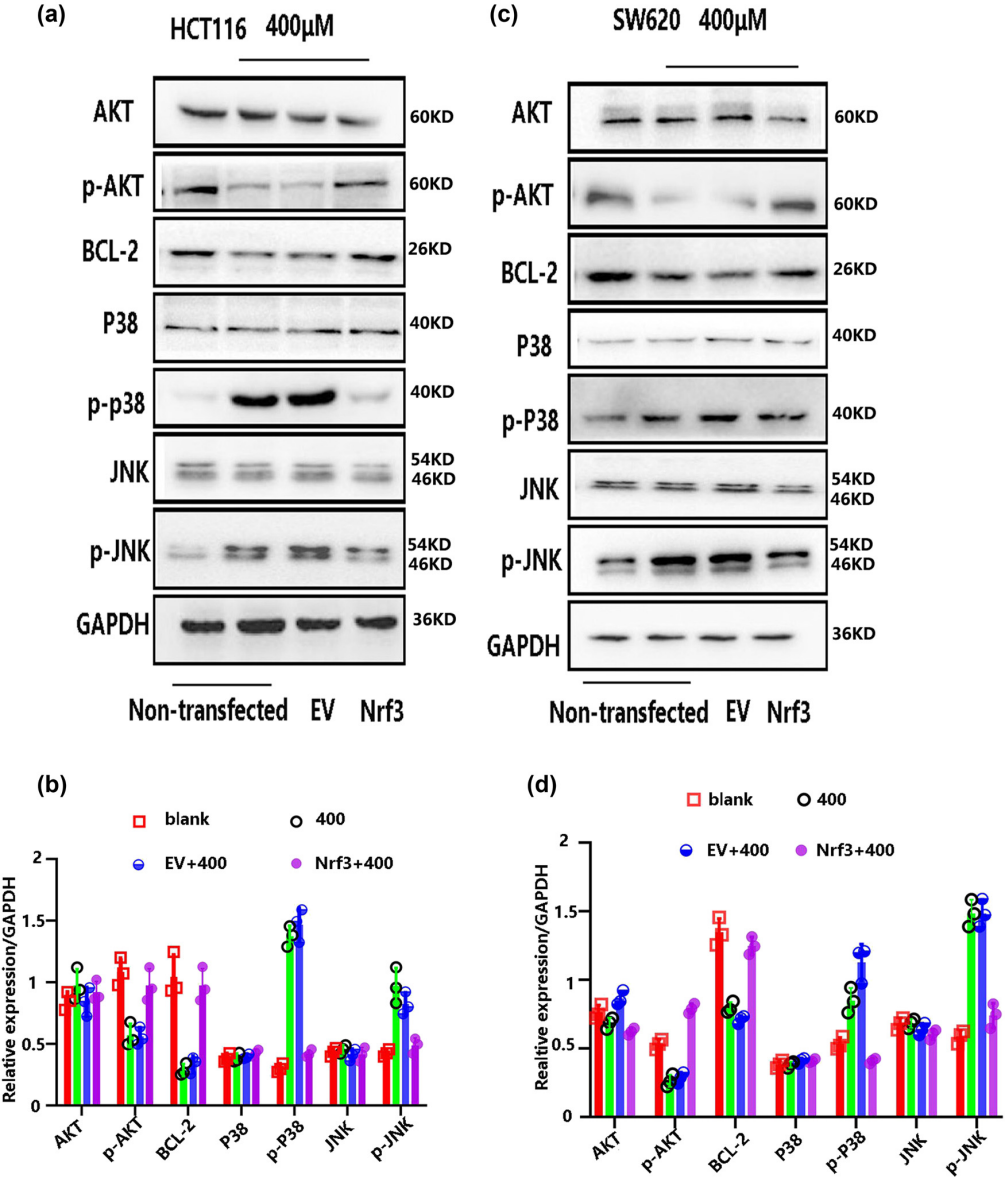
The influence of Nrf3 on oxidative signals in HCT116 and SW620 cells following a 12-h treatment with H_2_O_2_. (a and b) The influence of Nrf3 on oxidative signals in HCT116 cells under H_2_O_2_-induced oxidative stress (at a concentration of 400 μM). (c and d) The influence of Nrf3 on oxidative signals in SW620 cells under H_2_O_2_-induced oxidative stress (at a concentration of 400 μM).

The findings indicated that the upregulation of Nrf3 resulted in enhanced expression of p-Akt and Bcl-2, while concurrently reducing the expression of p-JNK and p-P38 following a 12 h exposure to elevated concentrations of H_2_O_2_ (400 μM) in both HCT116 (Figure 5a and c) and SW620 cells (Figure 5c and d).

### 3.6 Nrf3 decreased 5-FU-induced colon cancer cell death

Oxidative stress plays a crucial role in the initiation and progression of tumors, and it also serves as a significant mechanism for cancer treatment, including radiotherapy and certain chemical drugs (5-Fu and cisplatin). Given that Nrf3 enhances cell survival during oxidative stress induced by H_2_O_2_, we aimed to investigate whether Nrf3 reduces the susceptibility of cancer cells to these drugs. Consequently, we focused on further studying the effects of 5-Fu.

**Figure 6:**
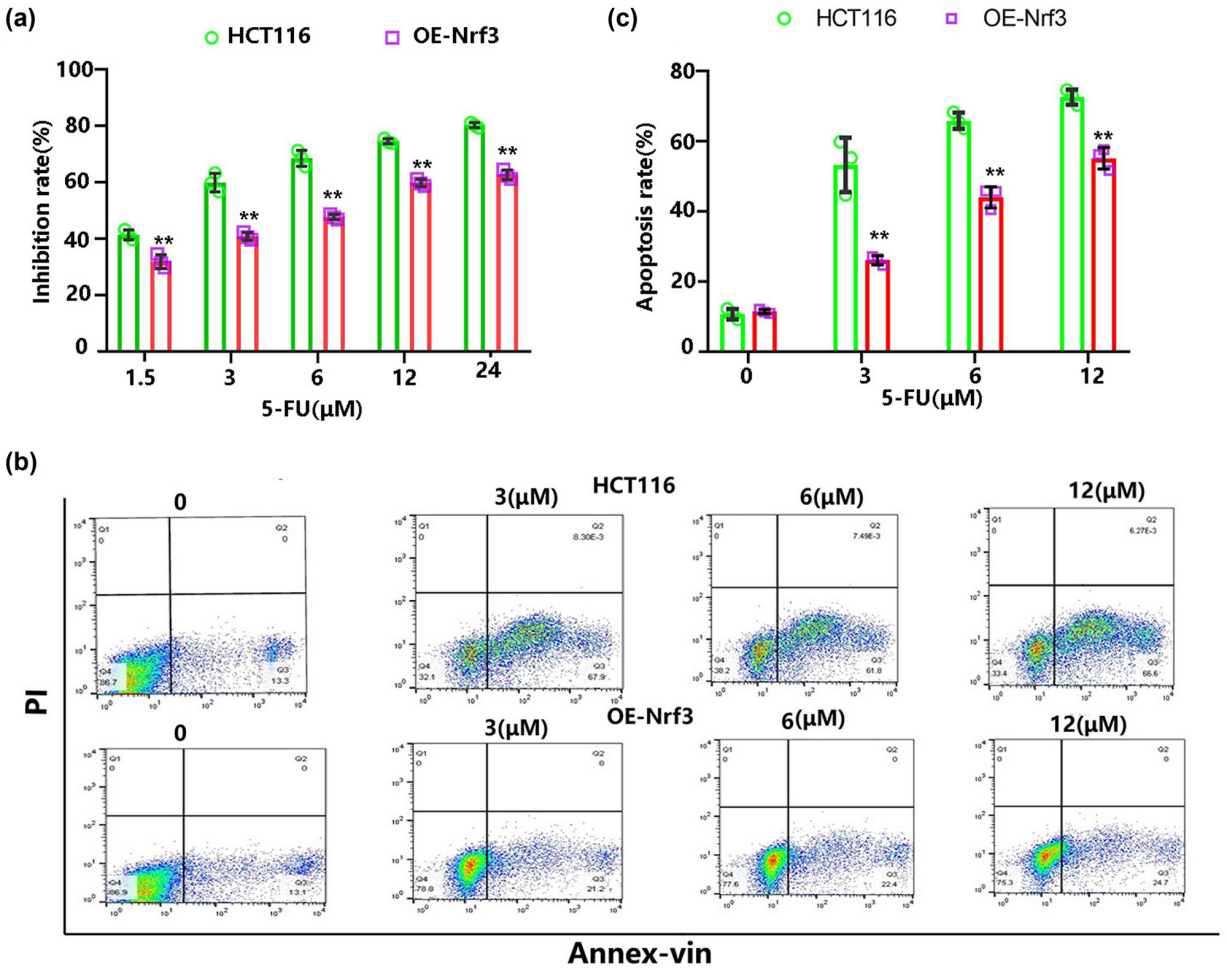
Nrf3 decreased 5-FU-induced death in HCT116 cells. The cells were exposed to varying concentrations of 5-FU for a duration of 24 h. (a) Nrf3 exhibited a reduction in the inhibitory effects caused by 5-FU treatment. (b) The representative flow cytometry graph depicting cellular apoptosis. (c) Quantification of the apoptosis rate in the cells.

Cells were cultured and exposed to varying concentrations of 5-Fu for a duration of 24 h. Subsequently, cell viability was assessed using CCK-8, while apoptosis was measured using flow cytometry. The findings indicated that Nrf3 exhibited a positive impact on cell viability (Figure 6a) and a negative effect on apoptosis (Figure 6b and c).

## 4 Discussion

In the present study, Nrf3 has been demonstrated to play a crucial role in the survival of colon cancer cells under oxidative stress through the activation of the Akt/bcl-2 signaling pathway. Initially, it was observed that Nrf3 expression was noticeably increased following treatment with H_2_O_2_. Furthermore, the overexpression of Nrf3 was found to significantly enhance cell viability and reduce cell apoptosis *in vitro*, whereas the knockdown of Nrf3 yielded contrasting outcomes. Additionally, Nrf3 was found to decrease the levels of ROS and MDA after H_2_O_2_ treatment. The potential mechanisms underlying Nrf3’s promotion of colon cancer cell survival may be associated with the Akt/Bcl-2 pathway. Ultimately, our investigation revealed that the augmentation of Nrf3 led to a reduction in the occurrence of stress-related chemical-induced (5-Fu) cellular demise, consequently leading to the development of resistance towards the administered drug.

Accumulating evidence displayed that an increase in Nrf3 is observed in colon cancer [20], prostate cancer [26], and hepatocarcinoma [25]. However, the mechanism underlying Nrf3 upregulation remains elusive. Considering that oxidative stress can lead to carcinogenesis, we examined whether Nrf3 expression is affected by a redox imbalance. Our results revealed that Nrf3 expression increased after H_2_O_2_-induced oxidative stress in colon cancer cells (Figure 1a–d). Furthermore, we found that over-expression of Nrf3 in colon cancer cells promoted cell proliferation and survival (Figure 2a) and decreased cell apoptosis (Figure 2c) with H_2_O_2_ treatment. In addition, our results showed that Nrf3 decreased ROS and MDA levels after H_2_O_2_-induced oxidative stress in colon cancer cells (Figure 3a–d). According to these data, Nrf3 expression may be correlated with oxidative stress levels.

Multiple signaling pathways [24,27] control oxidative stress-induced cell death, including signals that promote cell survival (e.g., Akt, bcl-2, NF-κB, and ERK) and signals that induce cell death (e.g., p38 and JNK). To investigate the potential mechanisms underlying the decrease in cytotoxicity of oxidative stress caused by Nrf3, we initially examined several cell signals (Akt, Bcl-2, p38, and JNK) that have been reported to play a significant role in oxidative stress in cancer. We observed that high levels of H_2_O_2_-mediated oxidative stress (200 and 400 μM) reduced the activity of the Akt/bcl-2 pathway and increased the expression of p-P38 and p-JNK in colon cancer cells, but there is no influence on these signals at the low level of H_2_O_2_ (100 μM) (Figure 4). The inhibition of phosphorylated Akt has been shown to have an inhibitory effect on various downstream pro-survival signals and antioxidant enzymes, including NF-κB and ARE pathways. Therefore, the increased activity of Akt/bcl-2 in colon cancer cells may play a crucial role in promoting cell survival under conditions of high oxidative stress. Additionally, JNK and p38, which are often activated through similar signaling pathways, can activate several pro-death pathways, such as the subsequent activation of Bid or the release of cytochrome c and AIF.

What are the underlying mechanisms responsible for the participation of Nrf3 in reducing the cytotoxic effects of H_2_O_2_-induced oxidative stress? Nrf3 [28–30] also plays a role in the signaling pathways involved in the progression of cancer. For instance, Nrf3 [31] promotes the proliferation of human hepatocellular carcinoma (HepG2) cells through the Wnt/β-catenin pathway. Therefore, it is crucial to investigate the impact of Nrf3 on oxidative signaling to elucidate the mechanisms by which Nrf3 inhibits cell death induced by H_2_O_2_. Our findings demonstrate that upregulating Nrf3 expression mitigates the inhibition of Akt/bcl-2 by H_2_O_2_ in colon cancer (Figure 5). Despite the absence of discernible effects of Nrf3 on p38 and JNK, it is noteworthy that Nrf3 overexpression significantly impeded the expression of p-P38 and p-JNK during high levels of H_2_O_2_-induced cellular demise (Figure 5). Our findings imply that the reduction in H_2_O_2_-induced cytotoxicity by Nrf3 is associated with the activation of Akt/Bcl-2 and the inhibition of p38/JNK.

The aforementioned findings suggest a strong association between the upregulation of Nrf3 expression in colon cancer cells and oxidative stress. This protein plays a crucial role in enhancing pro-survival signals (Akt and Bcl-2) while dampening pro-death signals (JNK and p38) during oxidative stress, ultimately resulting in a reduction in the cytotoxic effects of H_2_O_2_-induced oxidative stress. While this study does not rule out the possibility of other mechanisms contributing to the Nrf3-mediated inhibition of H_2_O_2_ cytotoxicity, it does offer potential implications for mitigating oxidative adaptation and increasing the sensitivity of cancer cells to stress-associated chemicals [10,32], such as 5-FU, cisplatin, and bortezomib. Consequently, 5-FU was chosen to validate this hypothesis. Our findings demonstrate that the upregulation of Nrf3 expression in colon cancer cells significantly diminishes the efficacy of 5-FU in inducing cell death (Figure 6). This research elucidates the novel role of Nrf3 as an inhibitor of H_2_O_2_-induced cytotoxicity, thereby offering potential avenues for investigating a novel strategy to overcome resistance in colon cancer cells to therapeutic interventions.
